# Mechanochromic and Thermochromic Sensors Based on Graphene Infused Polymer Opals

**DOI:** 10.1002/adfm.202002473

**Published:** 2020-05-19

**Authors:** Izabela Jurewicz, Alice A. K. King, Ravi Shanker, Matthew J. Large, Ronan J. Smith, Ross Maspero, Sean P. Ogilvie, Jurgen Scheerder, Jun Han, Claudia Backes, Joselito M. Razal, Marian Florescu, Joseph L. Keddie, Jonathan N. Coleman, Alan B. Dalton

**Affiliations:** ^1^ Department of Physics Faculty of Engineering & Physical Sciences University of Surrey Guildford GU2 7XH UK; ^2^ Department of Physics University of Sussex Brighton BN1 9RH UK; ^3^ Laboratory of Nano‐Optics and Organic Electronics Department of Science and Technology Linköping University Norrköping SE‐601 74 Sweden; ^4^ School of Physics CRANN and AMBER Trinity College Dublin Dublin 2 Ireland; ^5^ Advanced Technology Institute University of Surrey Guildford GU2 7XH UK; ^6^ DSM Coating Resins Sluisweg 12 Waalwijk 5145 PE The Netherlands; ^7^ Chinese Academy of Sciences CN‐36220 Quanzhou CN CN‐36220 Quanzh China; ^8^ Applied Physical Chemistry University of Heidelberg Heidelberg 69120 Germany; ^9^ Institute for Frontier Materials Deakin University Geelong VIC 3216 Australia

**Keywords:** colloidal crystals, mechanochromic sensors, pristine graphene, self‐assembly, time‐temperature indicators

## Abstract

High quality opal‐like photonic crystals containing graphene are fabricated using evaporation‐driven self‐assembly of soft polymer colloids. A miniscule amount of pristine graphene within a colloidal crystal lattice results in the formation of colloidal crystals with a strong angle‐dependent structural color and a stop band that can be reversibly shifted across the visible spectrum. The crystals can be mechanically deformed or can reversibly change color as a function of their temperature, hence their sensitive mechanochromic and thermochromic response make them attractive candidates for a wide range of visual sensing applications. In particular, it is shown that the crystals are excellent candidates for visual strain sensors or integrated time‐temperature indicators which act over large temperature windows. Given the versatility of these crystals, this method represents a simple, inexpensive, and scalable approach to produce multifunctional graphene infused synthetic opals and opens up exciting applications for novel solution‐processable nanomaterial based photonics.

## Graphical abstract

Graphene‐infused photonic crystals are fabricated using evaporation‐driven self‐assembly of soft polymer colloids. The crystals can be deformed mechanically or can reversibly change color as a function of temperature. Their sensitive mechanochromic and thermochromic properties are demonstrated for application areas ranging from time‐temperature indicators to security devices.

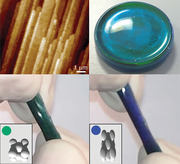

## 1 Introduction

Nature offers incredible examples of functional materials. Structural color, as found in butterfly wings or opal gem stones, is particularly fascinating.^[^ ^1^ ^]^ Mimicking such behavior using synthetic photonic crystals consisting of highly ordered assemblies of monosize colloidal particles^[^ ^2^, ^3^, ^4^, ^5^, ^6^, ^7^ ^]^ is a promising avenue for a range of novel and emerging applications.^[^ ^7^, ^8^, ^9^, ^10^, ^11^, ^12^, ^13^, ^14^, ^15^, ^16^ ^]^ One of the major limiting factors for the realization of colloidal photonic crystals requiring color perceptibility is their opaque nature. The origin of the opacity is structural disorder causing strong incoherent scattering that generates diffuse light in the presence of a low refractive index contrast. A possible method to counteract these issues is to embed nanomaterial dopants in the dielectric opal matrix.^[^ ^17^, ^18^, ^19^, ^20^, ^21^, ^22^, ^23^, ^24^ ^]^ For example, Aguirre et al.^[^ ^17^ ^]^ incorporated carbon black micro‐particles into poly(methyl methacrylate) (PMMA) to produce powdered pigments for coating applications. In other reports,^[^ ^19^, ^20^ ^]^ chromatic sensors were fabricated using core‐shell particles infused with sub‐50‐nm‐diameter carbon particles. More recently, Huang et al.^[^ ^25^ ^]^ fabricated photonic inks made of polystyrene‐based colloidal crystals containing graphene quantum dots. A hydrogel colloidal crystal made of silica nanoparticles and graphene oxide (GO) has also been reported for smart sensing and counterfeiting applications.^[^ ^26^ ^]^

A particularly interesting dopant candidate is graphene. Graphene has extraordinary electronic and optical properties.^[^ ^27^ ^]^ For instance, the high carrier mobility enables ultrafast extraction of photo‐generated carriers at significantly low light intensities leading to high‐bandwidth operation.^[^ ^28^ ^]^ Moreover, graphene has a wide absorptive spectral range from the ultraviolet to the infrared, with the optical absorption being proportional to the number of monolayers per nanosheet. Recent developments to produce liquid exfoliated, defect‐free pristine few‐layered graphene in large quantities have opened up new exciting avenues for applications.^[^ ^29^, ^30^ ^]^ However, incorporating pristine graphene into ordered polymer matrices in a controllable manner has proven rather challenging, and as a consequence, heavily functionalized graphene oxide (GO) is often used instead.^[^ ^31^, ^32^, ^33^, ^34^, ^35^, ^36^, ^37^ ^]^ Consequently, very little is known about water‐based functional composites enhanced with pristine graphene.

Guided by our work on fabricating ordered polymer colloid assemblies containing carbon nanotubes (CNTs),^[^ ^38^, ^39^, ^40^, ^41^, ^42^ ^]^ we have developed an evaporation‐driven self‐assembly method to fabricate highly ordered conducting polymer composites and photonic crystals containing pristine graphene.^[^ ^50^ ^]^ Using a polymer colloid template, a segregated distribution of graphene is achieved with periodicities comparable to the wavelengths of visible light. The resulting composites possess a low electrical percolation threshold. Furthermore, through careful control of the drying conditions, these composites form colloidal crystals which exhibit strong angle‐dependent structural color and a stop‐band that can be repeatedly shifted in the visible spectral range. We show that our opalescent colloidal crystals can be made to any size and thickness and can be crystallized on various substrates or be made freestanding. Importantly, the resulting photonic crystals (PCs) are free of cracks, and the stop band and mechanical deformability can be dialed in during the fabrication stage by changing the lattice parameters and the polymer glass transition temperature, respectively. Finally, we demonstrate the use of the graphene doped photonic crystals for a range of applications from strain gauges to time‐temperature indicators. Ultimately, this simple assembly process could be generalized to fabricate tunable crystal structures using a range of 2D crystals as the active filler, thus opening up a plethora of potential applications.

## 2 Results and Discussion

### 2.1 Evaporation‐Driven Self‐Assembly of Opal‐Like Graphene‐Enhanced Photonic Crystals

The polymeric latex used in this study produced through emulsion polymerization, is comprised of a random copolymer of methyl methacrylate (MMA), butyl acrylate (BA), and methacrylic acid (MAA) in the form of a charge‐stabilized colloidal suspension of polymer spheres. Polymer latex can undergo self‐ordering during sedimentation and compression under gravitational forces. Because of their highly ordered structure, distinct optical characteristics (photonic stop bands) can be achieved leading them to exhibit unique structural color. By carefully controlling the self‐assembly of the colloidal particles and taking into account the interplay between particle diffusion, inter‐particle forces, and settling dynamics,^[^ ^43^ ^]^ we achieved solids with long‐range particle ordering resulting in opal‐like crystals containing pristine graphene.

**Figure 1:**
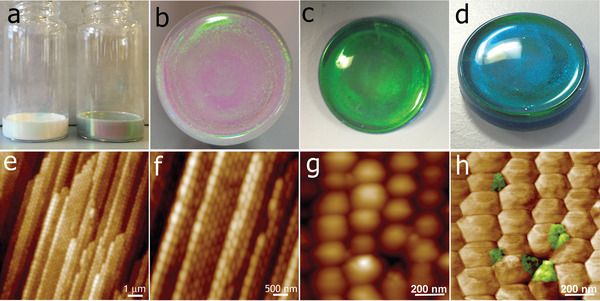
Optical images and internal microstructure of colloidal crystals enhanced with graphene. a) Photographs of wet colloidal dispersions used for photonic crystals (PC) (left) and with graphene for PC‐G (right). Representative photographs of fabricated colloidal photonic crystals: b) top‐view of a pristine PC; c) top‐view of a PC‐G; d) the same PC‐G when observed from a different viewing angle. AFM topographic images of e,f) PC‐G cross‐section showing the layered structure; g) height and h) phase images of the top surface of PC‐G showing graphene flakes (in false color) present in the interstitial sites.

**Figure** **1**a shows photographs of the initial wet dispersions with (right) and without (left) the graphene present used to assemble PCs. Optically, the graphene‐doped photonic crystals (referred to hereafter as PC‐G) have an intense green color under natural lighting conditions (Figure 1c) that gradually changes to a dark blue (Figure 1d) as the viewing angle is altered. In the PC‐Gs, the graphene is present at the interstitial sites between particles (Figure 1g,h). On the micrometer scale, the polymer particles assemble into hexagonal close‐packed (hcp) structures in well‐defined planes as shown on the cross‐sectional AFM images (Figure 1e,f). Simulations and experiments have found that the extent of crystalline ordering is affected by the relative times for water evaporation and crystal formation^[^ ^44^ ^]^ and disorder‐to‐order transitions^[^ ^45^ ^]^. These observed structures indicate that the evaporation rates are slow enough in our experiments to avoid jamming in a non‐crystalline structure.

**Figure 2:**
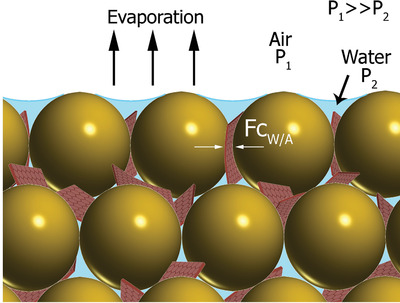
Proposed mechanism for the formation of highly ordered graphene networks. Schematic representation of the pressure difference across the surface of a layer of drying colloidal film and capillary forces acting on graphene during film formation.

As shown in our previous work,^[^ ^46^ ^]^ the capillary forces resulting from the receding water/air interface (*Fc,_W_*_/_ *_A_*) for the colloidal polymer system are ≈10^−7^ N, whereas the forces resulting from the proceeding capillary liquid bridge forces are one order of magnitude smaller (≈10^−8^ N). Graphene is present throughout the crystal formation process. During drying, the graphene is trapped at interstitial spaces between polymer particles and is subjected to the same capillary forces acting on the particles. As the film forms, at each contact surface between particles there is a localized distribution of both compressive and tensile stresses resulting from capillarity. In order to bend graphene flakes and conform them to the particle curvature, the superposition of forces must be large enough to overcome the graphene's resistance to bending (**Figure** **2**). Single‐layer graphene has the ability to conform to surface topography resulting from its low flexural rigidity.^[^ ^47^, ^48^, ^49^ ^]^ However, stacking graphene layers greatly increases flexural rigidity, indicating that thicker flakes will exhibit greater resistance to bending. Therefore, only highly exfoliated graphene flakes of up to a few layers (as used in this work) can be bent and efficiently fit at the particle boundaries and interstitial spaces. Additionally, the viscosity of the polymer near its glass transition temperature is very high and any flow is very slow.

The photonic crystals presented here are assembled using a technique we refer to as evaporation‐driven self‐assembly. During water evaporation, the top surface descends at a constant velocity, which will have the effect of accumulating particles at the air/water interface. Brownian diffusion re‐distributes the particles to diminish concentration gradients. Finally, sedimentation has the potential to accumulate particles at the bottom of the suspension in a highly ordered manner.

Following the arguments of Cardinal et al.,^[^ ^43^ ^]^ we use dimensionless numbers to determine which effect is dominant. To determine the relative importance of evaporation in relation to diffusion, we use the dimensionless Péclet number (*Pe*) which is written as

(1) $$Pe\;=\frac{EH_{0}}{D_{0}}$$

where *E* is the experimentally obtained water evaporation rate in units of velocity (*E* = 1.1 × 10^−7^ m s^−1^),^[^ ^50^ ^]^ H_0_ is the initial thickness of the suspension, and *D*_0_ is the Stokes–Einstein diffusion coefficient for the particles. For spherical particles, $D_{0}=\frac{kT}{6\pi \eta r}$, where *k* is the Boltzmann's constant, and *T* is the temperature, η is the viscosity of the liquid (water), and *r* is the particle radius. When *Pe* >>1, evaporation is dominant and particles will accumulate at the top descending interface, but for *Pe* <<1 diffusion will dominate and particles will be uniformly distributed during drying. The value of *Pe* for the polymer particles (*Pe_P_*) and for the graphene (*Pe_G_*) is high because of the large height (*H*_0_ = 0.5 cm) of the colloidal suspension. Using a standard approximation for the diffusivity of a 2D plate‐like particle with a diameter of 350 nm, we estimate *Pe_G_* to be ≈150, whereas for the 255 nm polymer particles, *Pe_P_* ≈320.^[^ ^51^, ^52^ ^]^

The settling velocity of spherical particles under gravitation at room temperature (*U*_0_) invoking the Stokes' drag force, is:^[^ ^43^ ^]^

(2) $$U_{0}=\frac{2}{9}\;\frac{\left(\rho_{\text{p}}-\rho_{\text{L}}\right)}{\eta }gr^{2}$$

where *ρ_p_* is the particle density (ρ_p_ = 1.1 g cm^−3^), *ρ_L_* is the liquid (water) density, and *g* is the gravitational acceleration. *U*_0_ for our 255 nm diameter polymer particles is calculated to be 3.5 × 10^−9^ m s^−1^. We find a distribution of diameters for the graphene platelet with the center of the distribution at 350 nm (see Figure S1, Supporting Information). Using this size, and considering the relatively high density of graphene, *U*_0_ is estimated to be 3 × 10^−7^ m s^−1^ for graphene flakes, which is considerably faster than the polymer particles.^[^ ^53^, ^54^ ^]^ The relative importance of sedimentation and evaporation is given by the sedimentation number, $N_{S}=\frac{U_{0}}{E}$. We find *N_S_* ≈ 0.03 for the polymer particles and *N_S_* ≈3 for the 350 nm graphene flakes. It is worth noting that this simple model ignores the effect of increased particle concentration and its associated effect on slowing the sedimentation velocity. Regardless, it is clearly valid in the initial stages of self‐assembly when the solids content of the system is still relatively low.

**Figure 3:**
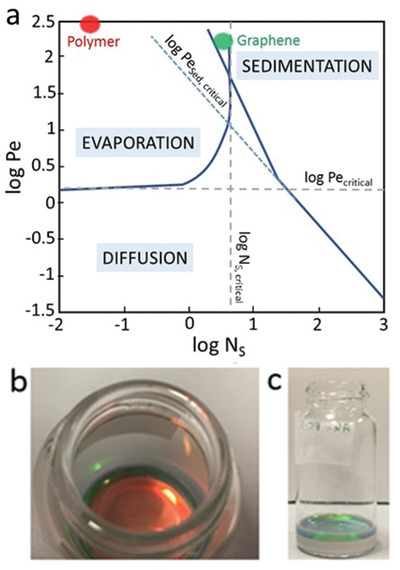
a) Drying regime map based on dimensionless coordinates Péclet number (*Pe*) and sedimentation number (*N_s_*) (Reproduced with permission.^[^ ^43^ ^]^ 2010, John Wiley and Sons). The red and green dots show the coordinates for the polymer particles and graphene, respectively, under the conditions used in the crystal formation. Photographs of the crystals forming during evaporation‐driven self‐assembly: b) top view, c) side view. The colored layer on the top is the assembled crystal, whereas the gray layer is the wet dispersion.

We invoke Cardinal et al.'s map of parameters (*Pe* and *N_S_*) to characterize the crystal formation process. As shown in **Figure** **3**, for the polymer particles, evaporation significantly dominates over both diffusion and particle sedimentation. In this regime, as the air/water interface descends during evaporation, the top of the film sweeps up the polymer particles, accumulating them at the top. In this mechanism, the colloidal crystal grows from the top downward in a self‐stratifying layer, which is clearly visible in the images in Figure 3b,c. As the surface of individual colloidal polymer particles is enriched in methacrylic acid (MAA), the negatively‐charged carboxylic acid groups lead to repulsion which acts to improve polymer particle ordering during the evaporation step,^[^ ^55^ ^]^ while the partial break‐up of the particle membranes and subsequent partial chain interdiffusion leads to the mechanical robustness of the crystals.^[^ ^56^, ^57^ ^]^

As shown on Figure 3, a 350 nm graphene particle will be in an intermediate mixed regime where both evaporation and sedimentation are dominant. Larger graphene flakes will predominantly sediment during the crystal formation, whereas smaller flakes will accumulate at the top and be mixed with the polymer particles in the colloidal crystal.

The crystal formation occurs from the top down. As such, it can be considered to be a 1D process. Larger area crystals can be made using containers with a larger area. The formation of the crystals is dominated by the competition between gravity, diffusion, and evaporation acting in the direction normal to the top surface. Hence, the scaling still holds. With a larger area, edge effects are minimized.

It is worth noting that conventional sedimentation of particles under gravity in a closed system without evaporation^[^ ^58^ ^]^ is ineffective for the fabrication of colloidal crystals containing graphene. Even few layer graphene sheets sediment out significantly faster than the polymer particles do. As a result, phase separation and formation of black sediment at the bottom of the vial occurs (Figure S1, Supporting Information).

### 2.2 Optical Properties of Graphene‐Enhanced Photonic Crystals

**Figure 4:**
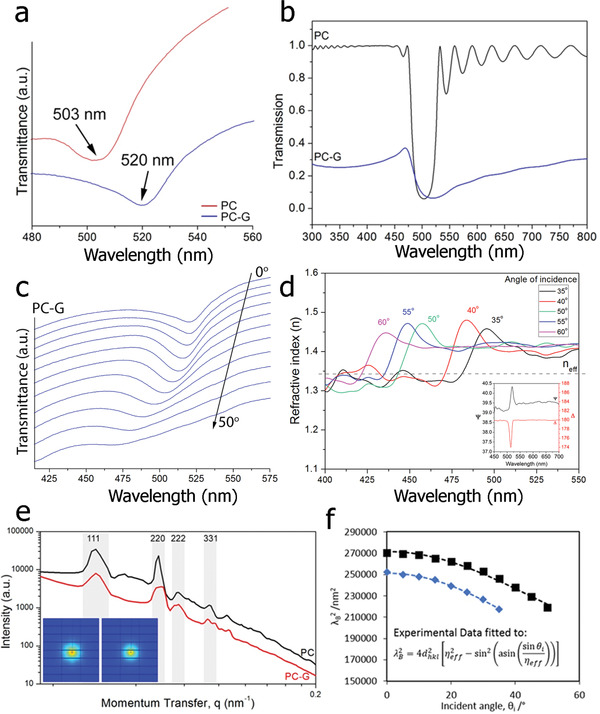
Optical properties of photonic crystals. a) Transmittance as a function of wavelength obtained at θ = 0° for a PC and PC‐G, showing significant red‐shifting of the stop‐band due to the inclusion of graphene. b) Simulated transmission from a pristine opal sample (black curve), an infiltrated opal sample with graphene spherical inclusions of radius 35 nm (blue curve). The thickness of the samples is 4000 nm. c) Variation in the transmission spectra with the angle of light incidence for the PC‐G. d) Pseudo‐refractive index *n*(λ) of PC‐G obtained through inversion of the ellipsometric data at different angles of light incidence. For comparison, dotted line shows the *n*_eff_ obtained as shown in Figure f (inset: Ellipsometric parameters *Ψ(λ)* and *Δ(λ)* measured at an angle of incidence of 20°). e) cSAXS data for the PC (black line) and PC‐G (red line) with insets showing the diffraction rings. f) Experimental (squares and diamonds) and simulated (dashed black and blue lines) Bragg wavelengths, λ_B_ for the PC (blue diamonds) and PC‐G (black squares). The data are fitted using a linear least squares regression to the equation shown in the inset (where *d*_hkl_ is the interplanar spacing, *n*_eff_ is the effective refractive index and θ is the angle of incidence).

The PC‐Gs possess the necessary ordering to satisfy the Bragg condition, and hence exhibit a distinct stop band positioned at approximately twice the particle diameter (≈520 nm), which is shifted upward by 17 nm with respect to the pristine crystal, as is shown in **Figure** **4**a. As shown in Figure 1a, visually, the PC appears milky white with a faint tint of green, most likely due to the undesired scattering of light.

The fitting of the Bragg equation shown in the inset of Figure 4f to the data allows for the calculation of the effective refractive index, *n*_eff_. From the resulting analysis, the *n*_eff_ of PC and PC‐G crystals are 1.26 ± 0.01 and 1.34 ± 0.01, respectively, which are in reasonable agreement with the *n*_eff_ values obtained using other methods as described in the Supplementary Information 1. Additionally simulations calculating the stop band positions (Figure 4b) of both the PC and the PC‐G are in good agreement with the experimental spectra (Figure 4a), even though the simulations consider only 4 µm thick PC‐G sample, while the fabricated crystals are as thick as 5 mm. Data obtained from coherent small angle x‐ray scattering (cSAXS) (Figure 4e) indicates that the lattice constant is 239 ± 2 nm for the PC and 240 ± 4 nm for the PC‐G. As the lattice constant is nearly identical for both types of crystals, it is likely that the inclusion of graphene is responsible for the redshift of the stop band by increasing the refractive index contrast^[^ ^59^ ^]^ in a unique combination with its wide spectral absorbance in the visible range^[^ ^60^ ^]^ relative to the undoped PC. Because the loading levels of graphene are extremely low, the presence of the graphene platelets has a minimal effect on the polymer particle ordering and on the periodicity of the crystal, but the concentration is sufficient to enhance the photonic effect. Similarly to natural opal gem stones^[^ ^61^ ^]^, all of the colloidal crystals are filled with interstitial water (≈9% by weight) (Figure S3, Supporting Information). Water that diffused into the polymer crystal forms nanometer sized domains of heterogeneous water. Such water domains possess a different refractive index than the polymer particles and hence acts as light scattering centers.^[^ ^62^, ^63^ ^]^

In natural opals, various internal imperfections give rise to incoherent scattering events and part of the transmitted spectrum is diffusely reflected. In the presence of graphene, the likelihood of absorption of the diffuse transmitted light is strongly enhanced as the incoherent scattering increases its effective optical path inside the opal. Hence, the parasitic reflections are reduced and the light Bragg‐scattered by the stop band dominates the reflection spectrum.

In order to characterize the optical reflectivity and confirm the modifications to the refractive index in the presence of graphene, spectroscopic ellipsometry was performed at angles‐of‐incidence, θ ranging from 20° to 60°. As the PC has very weak reflectivity, it was not possible to obtain a spectrum. For the PC‐G, representative ellipsometric spectra, showing Ψ (the ratio of the amplitude change of the *p‐* over the *s‐* polarization) and Δ (corresponding difference in phase changes) as a function of wavelength at θ = 20°, are presented in the inset of Figure 4d. A strong peak in both ellipsometric angles is observed in the wavelength range from 500 to 530 nm, explained by the reflections taking place at periodic interfaces of polymer particles in the colloidal crystal. In the remaining part of the spectral range, where the Bragg condition is not fulfilled, Ψ and Δ remain nearly constant. As is expected, when increasing the angle‐of‐incidence (measured with respect to the normal of the sample surface), the resonance peak is shifted toward shorter wavelengths (Figure 4c,d).

It should be noted that if a polymer with a different particle diameter is used for the fabrication of photonic crystals, their stopband will be directly correlated to the size of the particles which allows further tuning of their optical properties (Figure S2, Supporting Information).

### 2.3 Mechanochromic and Thermochromic Response of Photonic Crystals

Interestingly, due to the recoverable deformability of the crystals, notable changes in the position of the stop band can be achieved by mechanical modulation using lateral compression, stretching, in‐plane pressure or bending. The crystals are mechanically robust but can be deformed cyclically with no hysteresis in their performance (Movie S1, Supporting Information). At the same time, the stop band of the deformed PC‐G can be controlled mechanically, and a significant blueshift or redshift is observed, depending on the type of deformation applied. The ability to tune or modulate the optical properties makes the PC‐Gs attractive candidates for a wide variety of sensing applications with the output directly observable by the naked eye.

**Figure 5:**
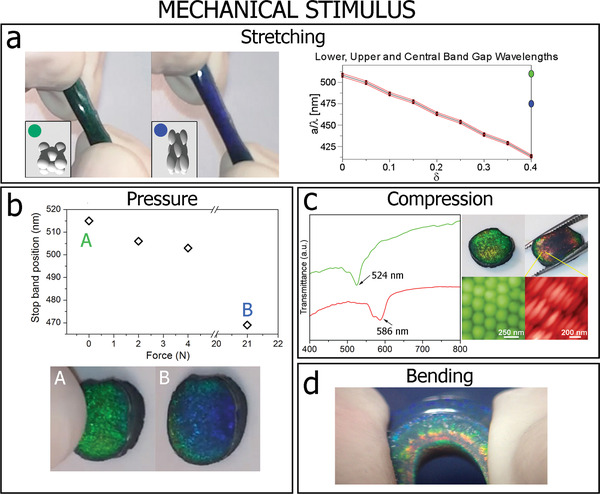
Variation in crystal morphology with deformation and the associated change in the stop band position. a) Deformation of stretchable PC‐G before (green) and during (blue) 150% elongation (Insets: Schematic representation of the variation in crystal morphology and the associated simulated change in the stop band position as a function of strain (Section S4, Supporting Information). b) Blue‐shifting of the stop band as a function of applied load. A corresponds to the PC‐G crystal before and B after the load was applied. c) Transmittance spectra for the PC‐G showing the red‐shift of the stop band when the crystal is subjected to an in‐plane compression. Optical photographs showing the PC‐G before and during macroscopic compression with corresponding AFM topographic images of microscopic particle deformation. d) Digital photograph of a PC‐G subjected to bending.

For example, the stop band of a stretched colloidal crystal shifts to shorter wavelengths as a result of a decrease in the spacing parallel to the crystal surface with increasing extension ratio. Consequently there is a visible change in the sample color from green to blue. As shown in Movie S1, Supporting Information, when the stress is released, the sample returns to its original shape. A schematic representation of the deformation of a crystal lattice is shown in the inset in **Figure** **5**a together with an associated simulated change in the stop band as a function of strain. Additionally, the stop band of a PC‐G gradually blueshifts under the application of contact pressure resulting in a consequent visual gradual color change from green to blue (Figure 5b; Movie S1, Supporting Information). This characteristic suggests a possible application of the PC‐Gs as a sensor to be used in fingerprint detection providing a multi‐channel response (with pressure and time). As shown in Figure S3, Supporting Information, pressing the crystals with a bare finger can reveal fingerprints with high precision showing well‐defined ridges from the skin.

By applying an in‐plane uniaxial compression, a significant redshift of the stop band of ≈62 nm is observed in the transmittance spectra since the compression causes a decrease of spacing parallel to the crystal surface (Figure 5c). Although, not visible in the image, the interparticle distance in the cross‐section plane always increases due to the volume conservation of the individual colloidal particles. But, when the stress is released, the sample returns to its original shape. Bending of the PC‐G crystals results in a rainbow‐like color variation along the cross‐section effectively producing a microscopic 2D strain field (Figure 5d). This mechanochromic response of the PC‐Gs is determined by the affine deformation of the particles under stress with the percentage change in stop band wavelength equal to the percentage strain. This corresponds to a sensitivity related by the initial stop band wavelength Δλ/ε(%) = λ_0_/100 = 5.2 nm/%, which is verified experimentally for the applied strain and wavelength shift above. This sensitivity is competitive with mechanochromic sensors reported in the literature^[^ ^64^, ^65^ ^]^ and could be further increased by modifying the size of the latex particles and thereby the initial stop band position. Bending of the PC‐G crystals results in a rainbow‐like color variation along the cross‐section, effectively producing a microscopic 2D strain field that is related to varying degrees of particle deformation from top to bottom (Figure 5d).

The crystals can also act as smart shape‐memory polymers that can memorize and recover their shape and color after experiencing an external stimulus, for example, heat (Movie S2, Supporting Information). The temperature of PC‐G crystals can be repeatedly shifted above and below their glass transition temperature (*T*_g_) value. Each time the crystal experiences elevated temperatures, it gradually relaxes back to the initial shape configuration while at the same time exhibiting gradual structural color change as the stop band returns to its original value pre‐deformation. This striking thermochromic effect of the crystals can be used for non‐invasive characterization of shape‐memory effects.

**Figure 6:**
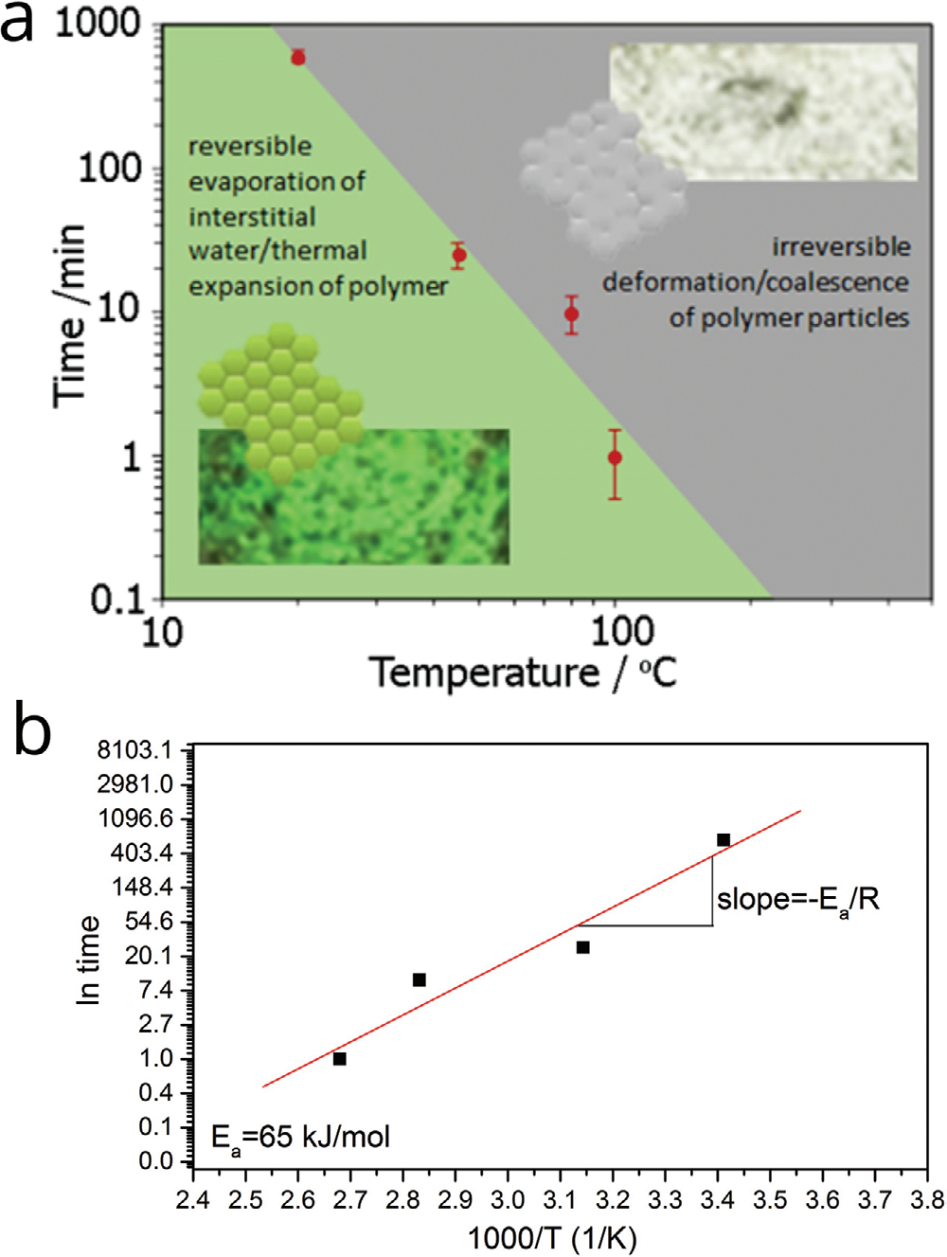
Graphene based colloidal photonic crystals for time‐temperature indicator applications. a) Time versus temperature plot showing regimes at which the interfacial structural transitions occur, resulting in an associated color change. Inset images: Optical photographs of the PC‐G crystal and schematic representation of particle boundaries showing the transition of color from green to transparent. b) Determination of activation energy for diffusion for the PC‐G crystals.

Very interestingly, the PC‐Gs can be used as time–temperature indicators (TTI) for intelligent packaging to offer a visual indication of whether perishables, such as food, pharmaceuticals, chemicals, inks, paints or coatings have experienced undesirable time‐temperature histories. If the crystals are subjected to elevated temperatures, they act as sensitive visual TTIs that function over a broad temperature range (from room temperature to 100 °C). The crystals contain up to 10% of water locked within their crystalline structure, and when they are exposed to atmospheric conditions, they lose their distinctive green color turning either transparent or opaque (associated with the increase in light absorption with the crystal's thickness) (Figure S4, Supporting Information). The distinct but gradual color change with time is associated with the loss of refractive index contrast when polymer/polymer boundaries are formed when intervening water is lost. The thermal expansion of polymer particles and interfacial water (both increasing the lattice constant) with increasing temperatures produces a redshift of the stop band, which is extremely sensitive to even a small rise in temperature. A time–temperature phase diagram (**Figure** **6**d) shows the combinations of time and temperature at which the interfacial structural transitions occur, resulting in an associated stop band shift and consequent gradual color change. The shaded green region in the figure indicates the conditions at which the TTIs exhibit reversibility. It means that if the crystals are rehydrated, their color returns to the original green. In this regime, they are operating close to their minimum film formation temperature (MFFT)—the lowest temperature at which they will experience complete coalescence; hence the particle deformation is incomplete and particle–particle interfaces still exist.^[^ ^56^ ^]^

Because of the presence of a network of pores as well as hydrophilic functional groups at the particle boundaries, the water diffusion will proceed along the interstitial sites allowing for good permeability.^[^ ^63^, ^66^ ^]^ The gray‐shaded region in Figure 6a indicates that at higher temperatures or longer exposure times, there is a certain cut‐off point where the crystals lose their color irreversibly with the stop band completely disappearing. In this region, the diffusion of individual polymer chains across particle−particle boundaries results in irreversible and complete coalescence of the particles, which is a well‐known occurrence in polymer latex films.^[^ ^67^ ^]^ The periodicity disappears, and thus Bragg's diffraction does not apply anymore. The loss of the stop band can be treated as a diffusion driven process where the time for the transition is defined by the time needed for the polymer chains to diffuse across the interfaces between particles.

When developing a visual monitoring TTI device to maintain product quality it is essential that the TTI accurately mimics the product's kinetic reactions related to the product's shelf‐life defined by the Arrhenius equation^[^ ^68^ ^]^ written as

(3) $$k=Z\exp^{-\frac{E_{a}}{RT}}$$

where *k* is the reaction rate constant, *Z* is a temperature independent pre‐exponential factor, *E_a_* is the activation energy describing the temperature sensitivity of the quality loss reaction, *R* is the universal gas constant. Briefly speaking, it is important that the activation energy of the TTI device does not differ by more than 20 kJ mol^−1^ from the activation energy of the product of interest.^[^ ^69^ ^]^

In our case, the irreversible nature of the TTI is related to the complete coalescence of particles during thermal reactions, while coalescence of particles requires the diffusion of polymer chains a distance on the order of their radius of gyration, *R_g_* across particle interfaces (where *R*_g_ is in the polymer melt). The diffusion coefficient (*D*) is related to the distance of diffusion, *x* and the time, *t* as

(4) $$D\sim \frac{\left\langle x^{2}\right\rangle }{t}\;\sim \;\frac{R_{\text{g}}{}^{2}}{t}$$

Equation (4) implies that the time for the chains to diffuse their radius of gyration *R_g_* is given as ≈$\frac{R_{g}^{2}}{D}$. Important for this model is the concept that diffusion is thermally activated and that temperature significantly affects *D* and hence can be described by the Arrhenius relationship of the form:

(5) $$D\;=\;D_{0}\exp\left(\frac{-E_{a}}{RT}\right)$$

where *D*_0_ is a constant for a given diffusing system (m^2^ s^−1^). Substituting in for *D* we see that:

(6) $$t\sim \frac{R_{\text{g}}{}^{2}}{D_{\text{o}}}\exp\left(\frac{E_{a}}{RT}\right)$$

Hence, plotting the natural logarithm of the time (*ln t*) that is required to achieve optical clarity and irreversible coalescence against the reciprocal temperature of the experiment, then there is a linear relationship

(7) $$\ln t\;\approx \;ln\;\frac{R_{g}^{2}}{D_{o}}\;+\;\left(\frac{E_{a}}{RT}\right)$$

and hence the activation energy for diffusion can be obtained from the gradient in Figure 6b.

The activation energy found for PC‐G is 65 kJ mol^−1^, which falls within the range of *E_a_* of food‐related chemical reactions leading to its spoilage and pathogenic microbial growth, which is between 30 and 140 kJ mol^−1^.^[^ ^70^ ^]^

### 2.4 Conducting Composites

Composite formation is achieved through direct mixing of surfactant stabilized aqueous dispersions of pre‐exfoliated graphene and the latex. The mixed dispersion is cast onto a substrate (such as glass slide or a free‐standing mold) and dried.

**Figure 7:**
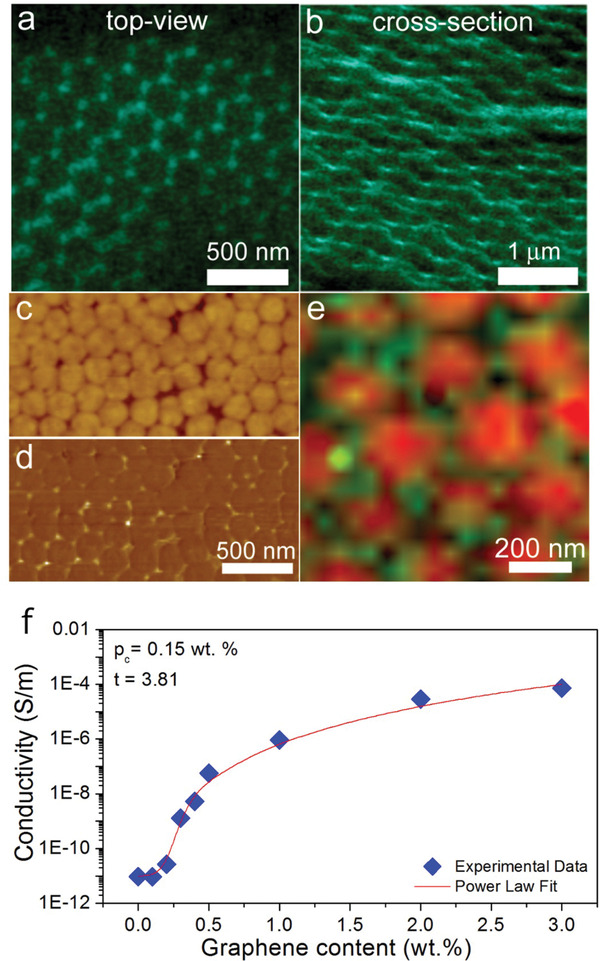
Hierarchically assembled graphene at the interstitial sites of highly ordered colloidal polymer matrix. SEM a) top view and b) cross‐section view of a composite containing 0.4 wt% of graphene showing petal‐like graphene arrangement. Corresponding AFM images showing c) topography and d) phase, respectively. e) Collage AFM/Raman map showing the composite's topography overlapped with the graphene 2‐phonon (2D) Raman band's (≈2700 cm^−1^) intensity. f) Electrical conductivity as a function of graphene content for graphene‐enhanced colloidal polymer composites.

As can be seen in **Figure** **7**a–d, during the composite assembly, graphene is present at the interstitial sites between the colloidal polymer particles, resulting in highly ordered 3D petal‐like graphene assemblies emerging. The majority of pre‐exfoliated graphene flakes used in this study have thicknesses between one and five layers with lateral sizes ranging from ≈100 to ≈2 µm (Figure S5, Supporting Information). By overlaying an atomic force microscope (AFM) height image and the Raman spectroscopy map of the two‐phonon 2D band (≈2700 nm^−1^) of the same sample area, it is clear that graphene is only positioned at the polymer's interstitial sites as shown in Figure 1e and a representative Raman spectrum in Figure S6, Supporting Information. The composites exhibit a characteristic percolation behavior (Figure 7f), with an electrical percolation threshold of ≈0.15 wt%, which is much lower than reported by others for reduced GO and pristine graphene, respectively in more conventional isotropic composites.^[^ ^31^, ^32^, ^71^ ^]^ A significantly higher critical exponent of 3.81 than the most accepted universal value of *t* = 2 can be attributed to the tunneling conduction previously witnessed in other composite systems.^[^ ^72^ ^]^

To understand the segregated composite formation mechanism, it is necessary to examine the deformation of polymer particles during the film formation process. In the first stage of drying, evaporation of water leads to the reduction of average inter‐particle distance, forcing colloidal particles into a hexagonal close‐packed (HCP) configuration.^[^ ^73^ ^]^ Existing models^[^ ^74^, ^75^ ^]^ show that there is a negative capillary pressure associated with the curved meniscus of water between the particles at the top surface. This negative pressure pulling particles together exerts compressive forces on the particles in the packed array below. At later stages of drying, the models show that pendant water present at the contacts between the particles presses them closer together, thereby leading to the elimination of interstitial voids. Finally, particles undergo coalescence through the inter‐diffusion of polymer chains across particle boundaries, ultimately forming a cohesive film.

It is interesting to note that, as produced, the pristine polymer latex has a glass transition temperature (*T*_g_) of 38 °C, while the *T*_g_ of the composite containing 0.4 wt% of graphene is reduced to 31 °C. As we have shown previously^[^ ^40^ ^]^, some of the surfactant can be adsorbed onto the nanomaterial surface, while the remaining free surfactant is confined in the interstitial spaces between the particles. Such free surfactant acts as a traditional plasticizer, softening the outer regions of polymer particles (mimicking core‐shell particles). It is likely that the softer polymer shell facilitates the formation of long‐range petal‐like ordering of graphene.

## 3 Conclusion

The study presented here provides the first experimental demonstration of mechanically robust, free‐standing, flexible, and thick synthetic opal containing pristine graphene locked in a colloidal polymer crystal lattice. The graphene‐enhanced colloidal crystals were, for the first time, fabricated using a novel inexpensive method of evaporation‐driven self‐assembly and have a range of applications as mechanochromic and thermochromic sensors. The presence of graphene enhances the structural color because of its wide spectral absorbance and higher refractive index. Importantly, the effects happens at significantly lower weight fractions compared to other carbon‐based fillers, such as carbon black. The versatile fabrication process can employ different particle sizes and glass transition temperatures, which allows property tuning. The color is responsive to pressure and stress, temperature and time and is fully lost when particles coalesce during exposure to high temperatures for prolonged times. These properties were demonstrated for application areas ranging from TTI sensors to security devices. The inclusion of a higher amount of graphene leads to the formation of conducting segregated network graphene‐based composites with a low electrical percolation threshold.

Ultimately, our method represents a generic way to assemble a broad range of 2D nanomaterials, such as transition metal dichalcogenide monolayers (e.g., MoS_2_, WS_2_), boron nitride sheets, etc. within the photonic crystals to achieve a plethora of potential novel sensing functionalities.

## 4 Experimental Section

##### Materials Fabrication

Graphite powder (2.5 g) purchased from Sigma Aldrich (product number 332461) was added to 100 mL of aqueous surfactant solution (0.5 mg mL^−1^ Triton X‐100) to give an initial graphitic concentration of 25 mg mL^−1^. This mixture was sonicated using a sonic tip (Sonics VX‐750 ultrasonic processor with flat head tip) for 4 h. The dispersion was left to stand overnight. The top 50 mL of the suspension was decanted into two 28.5 mL vials and centrifuged (Hettich Mikro 22R) for 90 min at 1500 rpm. The top 14 mL was then decanted into a 14 mL vial. The final concentration of graphene in water was 0.57 mg mL^−1^ as determined using UV–vis spectrophotometry.

The latex polymer used as a template for graphene assembly, provided by DSM Coating Resins (Waalwijk, The Netherlands), is based on a random copolymer of butyl acrylate (BA), methyl methacrylate (MMA) and methacrylic acid (MAA). The polymer particle size is 255 nm, its dry glass transition temperature (*T*_g_) is 38 °C, and the initial solids content is 55 wt%. The latex dispersion was prepared by semi‐batch emulsion polymerization.

Graphene‐surfactant dispersions prepared as described above were blended with latex by hand stirring and then homogenized by tip‐sonication in an ice‐cold water bath for 10 min. The final weight fraction of graphene in the composite dispersion relative to the polymer was 0.01 wt%.

To fabricate graphene‐latex composites, eight different concentrations of graphene in latex dispersion ranging from 0.01–3 wt% were prepared. Electrical properties of composites were investigated as a function of graphene concentration. A specially designed, gold‐plated ceramic substrate consisting of a large gold electrode in the middle and four smaller contacts (one on each side) was used. For measurements, polymer films were prepared by casting the composite dispersion using a metallic cube applicator with a nominal 150 µm gap width onto a ceramic substrate and dried for 24 h at room temperature. After drying, two 10 nm thick top gold electrodes of 3.5 × 3.5 mm area each were prepared by thermo‐evaporation of gold using an Edwards Evaporator. Dark current‐voltage characteristics have been obtained using a Keithley 487 picoammeter/voltage source. The final specific conductivity was calculated from the resistance and thickness of the film. All sample thicknesses were measured using a profilometer (Veeco, Dektak 8).

To grow photonic crystals, evaporation‐driven self‐assembly was successfully used. 2.5 mL of latex dispersion, containing 0.01 wt% of graphene, was left in an open glass beaker at room temperature for 4–6 days to induce the self‐assembly of the particles combined with drying of the suspension from above. The crystals were formed under a relative humidity of ≈65%. Since we have shown previously that the Triton X‐100 surfactant has a plasticizing effect on the polymer particles^[^ ^46^ ^]^, the same amount of surfactant was added to the latex dispersion for the fabrication of pristine PCs. This step was necessary to ensure that both, pristine and graphene‐enhanced PCs possess the same *T_g_*.

##### Materials Characterization

For topographic studies, an atomic force microscope (AFM) (NT‐MDT, Moscow, Russia), using semi‐contact mode, was employed. In order to study the cross‐section, the PCs were fractured in liquid nitrogen. In order to obtain AFM images of the crystals under deformation, the crystals were first immersed in hot water (80 °C) for 3 s, deformed and then quickly immersed in an ice cold water bath in order to “freeze” the structure for imaging.

Microstructural investigations of composite materials were carried out using a Hitachi S‐4000 scanning electron microscope (SEM) at an accelerating voltage of 7 kV. A latex dispersion containing 0.4 wt% of graphene was deposited a onto silicon dioxide/silicon wafer by drop casting and subsequently left to dry for 48 h at room temperature (≈23 °C). A sample was freeze‐fractured after immersion in liquid nitrogen, in order to investigate the cross‐sectional area. Samples were imaged without sputtering a metal onto their surface.

The *T_g_* of the latex composites was determined using a differential scanning calorimeter (DSC) (TA Instruments Q1000, New Castle, USA). Samples were deposited onto poly(tetrafluoroethylene) (PTFE) molds by drop casting and subsequently left to dry for 48 h at room temperature before being loaded into the DSC. A standard heating rate of 10 °C min^−1^ and cooling rate of 10 °C min^−1^ were used for all samples. The value of *T_g_* was taken in the first heating scan at the midpoint step‐wise increase of the specific heat associated with the glass transition.

The optical transmission measurements were carried out using a computer controlled double beam UV–vis spectrophotometer (Shimadzu UV2501PC dual‐beam spectrophotometer). The angle of incidence in the transmission measurement was changed from 0° to 55° by rotating the sample by means of a made‐in‐house sample holder. The absorption spectra were recorded from 200 to 900 nm.

In order to take the UV–vis spectra as a function of applied pressure, the crystals were immersed in hot water (≈80 °C) for 3 s, then placed into a glass beaker and a sample of known mass was placed on top of the crystals for 5 s. With the known weight still on top of the crystal, the beaker was filled with ice‐cold water in order to freeze the structure under compression. The crystal was then quickly transferred to the UV–vis spectrometer for measurement.

The standard ellipsometric quantities, Ψ and Δ which describe the changes in the amplitude and relative phase of the polarized light, respectively, were measured as a function of angle of incidence ranging from 20° to 55° at wavelengths ranging from 385 nm to 700 nm using a variable‐angle spectroscopic ellipsometer (J.A. Woollam Co., USA). For this measure, free‐standing photonic crystal samples were attached to the silicon dioxide/silicon wafer.

cSAXS (coherent small angle X‐ray scattering) experiments were performed at the Paul Scherrer Institute, Switzerland. A sample‐detector distance of 7160 mm (using a 7 m evacuated flight tube) and X‐ray energy of 8.9812 keV was used for measurements; the spot size was approximately 0.7 mm × 0.7 mm. The PILATUS 2M detector was used to capture scattering patterns from the mounted samples; this detector has 1475 × 1679 pixels which are 172 mm × 172 µm (an active area of 253.7 mm × 288.8 mm). Captured scattering patterns were integrated through the azimuthal angle to obtain radial scattering profiles.

## Conflict of Interest

The authors declare no conflict of interest.

## Supporting information

Supporting Information

Supporting Movie 1

Supporting Movie 2
