# Supplementary material for: Mechanochromic and Thermochromic Sensors Based on Graphene Infused Polymer Opals
Source: Adv Funct Mater. 2020 May 19;30(31):2002473. doi: 10.1002/adfm.202002473 (PMC7406018; doi:10.1002/adfm.202002473)
Supplement: Supplementary file 1 — Supporting Information [file ADFM-30-2002473-s001.pdf]

Copyright WILEY-VCH Verlag GmbH & Co. KGaA, 69469 Weinheim, Germany, 2020.

ADVANCED FUNCTIONAL MATERIALS

Supporting Information

for *Adv. Funct. Mater.,* DOI: 10.1002/adfm.202002473

Mechanochromic and Thermochromic Sensors Based on Graphene Infused Polymer Opals

Izabela Jurewicz, Alice A. K. King, Ravi Shanker, Matthew J. Large, Ronan J. Smith, Ross Maspero, Sean P. Ogilvie, Jurgen Scheerder, Jun Han, Claudia Backes, Joselito M. Razal, Marian Florescu, Joseph L. Keddie, Jonathan N. Coleman, and Alan B. Dalton**

Copyright WILEY-VCH Verlag GmbH & Co. KGaA, 69469 Weinheim, Germany, 2020.

Supporting Information

Mechanochromic and Thermochromic Sensors Based on Graphene Infused Polymer Opals

Izabela Jurewicz,* Alice A.K. King, Ravi Shanker, Matthew J. Large, Ronan J. Smith, Ross Maspero, Sean P.Ogilvie, Jurgen Scheerder, Jun Han, Claudia Backes, J. M. Razal, Marian Florescu, Joseph L. Keddie, Jonathan N. Coleman, Alan B. Dalton*

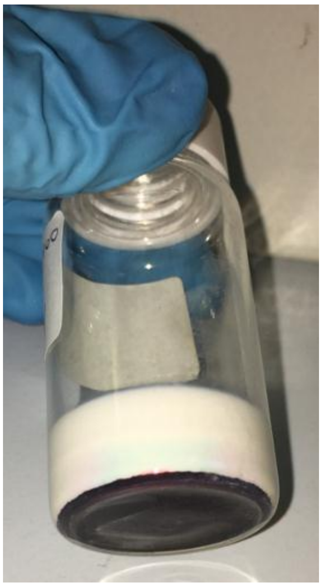

Figure S1. Gravitational sedimentation of the PC-G crystal showing phase separation of graphene and its precipitation on the bottom of the vial.

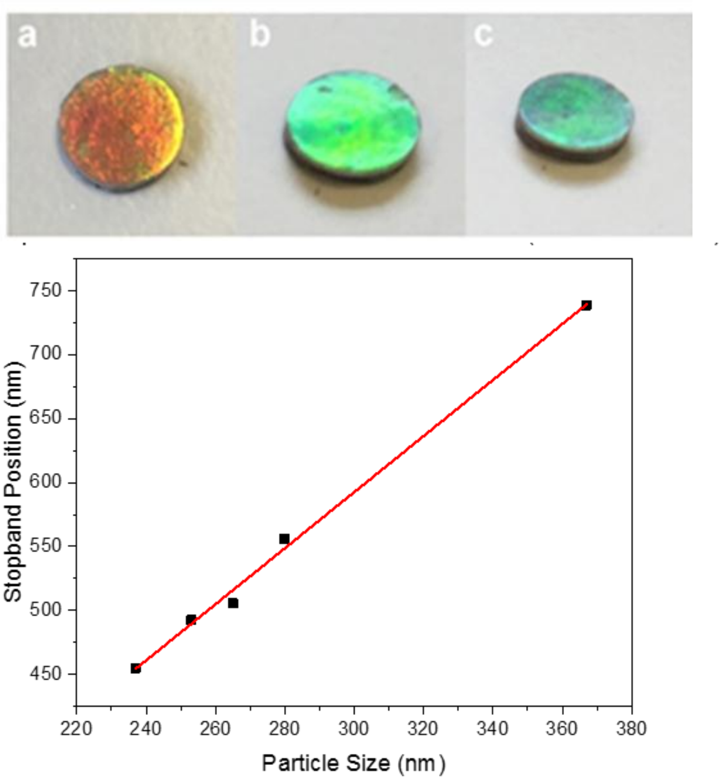

Figure. S2. (a-c) Digital photographs of photonic crystals made of 295 nm polymer particles containing 0.01 wt.% of graphene when observed from different viewing angles. (d) Plot showing the relationship between the stopband of the photonic crystal and the particle size.

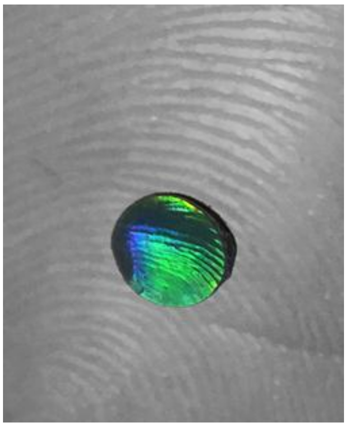

Figure S3. A photograph of fingerprint imprinted into a PC-G where ridges can be clearly seen.

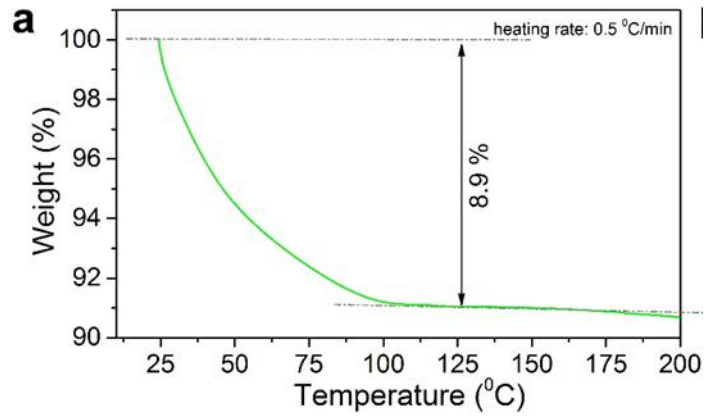

Figure S4. Thermogravimetric analysis (TGA) data showing the amount of water present within the PC-G.

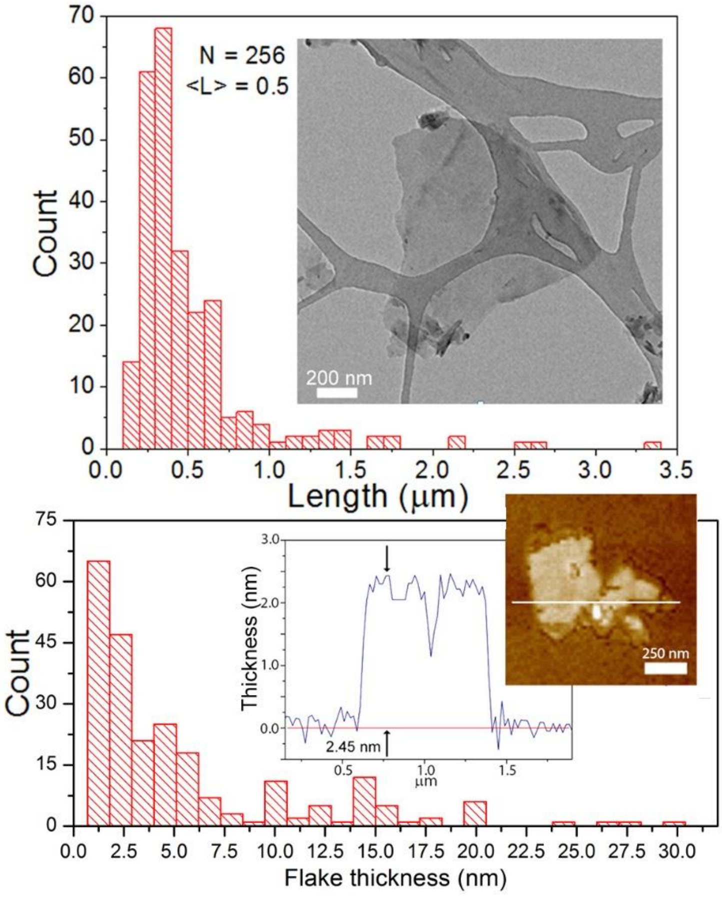

Figure S5. (top) Histogram of the size distribution of graphene flakes obtained by TEM. Inset: a representative TEM image of a graphene flake. (bottom) Graphene flake thickness histogram obtained by AFM. Inset: A representative zoomed image of graphene flakes and corresponding line scan taken horizontally through the image as marked with a white line. From this analysis, the topographic height of the graphene flake is measured to be about 2.45 nm. Considering that the apparent AFM thickness of a single layer of liquid exfoliated graphene is typically ~0.9 nm, the AFM histogram suggests the graphene sheets to be composed of only few-layers

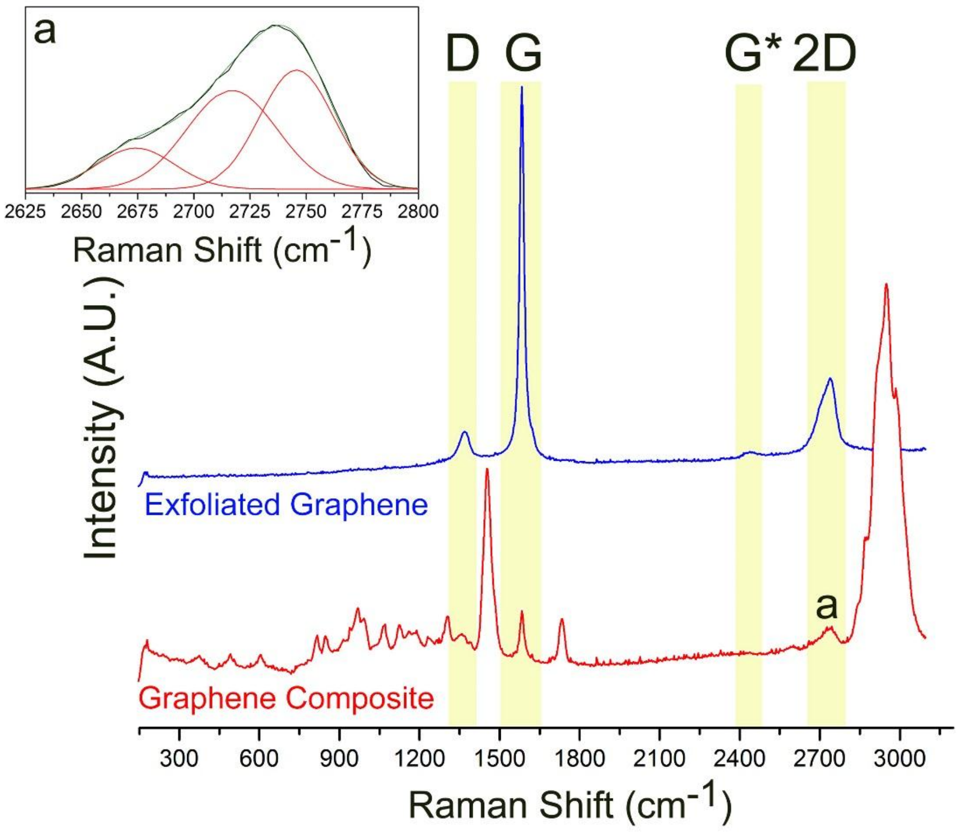

Figure S6. Raman spectra of exfoliated pristine graphene and the same graphene incorporated into a colloidal matrix.

Supporting Information SI 1.

Estimation of effective refractive index, n_{eff} of PC and PC-G.

METHOD 1 is shown in the main manuscript file.

METHOD 2: The effective refractive index, n_{eff} was also calculated by plotting λ_{111}^2versus $sin^2\theta$. The linear fit of the data was then used to calculate the n_{eff} for both PC and PC-G crystals. Fixed particle size *D* =239 nm and *D* =240 nm (as determined by x-ray scattering (cSAXS), for PC and PC-G respectively was used in the calculations. And the n_{eff} ,using this approach was calculated to be 1.33 and 1.28 for PC-G and PC respectively.

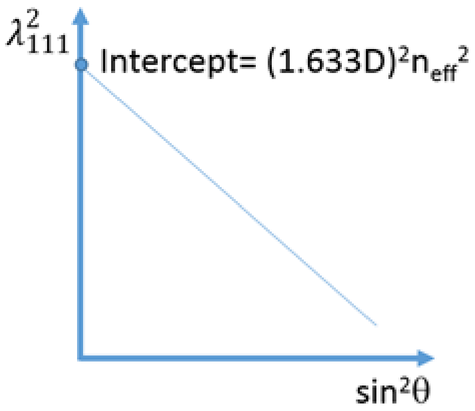

Figure S6. Square of the wavelength that has been diffracted from the (111) planes, λ_{111}^2 as a function of $sin^2\theta$ that can be used to determine the effective refractive index, n_{eff}.

METHOD 3: When Bragg condition

$$\lambda_{max} = 2d_{111}\sqrt{n_{eff}^2 - sin^2\theta} \qquad \text{(S1)}$$

(where $d_{111}=\sqrt{2/3}D$ is the lattice spacing of the (111) planes and D the particle size) is fulfilled, the effective refractive index, n_{eff} can be represented by the following equation:

$$n_{eff} = \left(n_p^2\phi_p + n_w^2\phi_w + n_G^2\phi_G\right)^{1/2} \qquad \text{(S2)}$$

where n is the refractive index and volume fraction (ϕ) of polymer particles (p), water (w) and graphene (G) respectively. To calculate n_{eff}, it was assumed that the colloidal crystals are made of hard incompressible spheres packed in regular hexagonal close-packed array (HCP) resulting in ϕ_p = 74%.

METHOD 4: Assuming that the reflection only takes place at the surface of the crystal, the ellipsometric parameters Ψ and Δ can be used to calculate an effective refractive index n_{eff} using the following equation derived from Fresnel's equations:

$$n_{eff} = \mathrm{Re}\left\{\tan\theta\sqrt{1-\frac{4\rho\sin^2\theta}{(\rho+1)^2}}\right\} \tag{S3}$$

where θ is the angle of incidence with respect to the surface normal, and ρ is the ellipticity, defined as $tan(\Psi)e^{i\Delta}$. The ellipticity represents the change in the polarization state of light after the reflection from the sample surface, across the spectral range. This equation is only valid for the wavelengths where the Bragg reflection is not satisfied.

The *n(λ)* increases from ~1.33 to ~1.41 as the wavelength increases, and a similar trend has also been observed by others for photonic materials. It should be noted that this analysis is only valid for the wavelengths where the Bragg reflection is not satisfied. The data are inverted without making any assumptions of the material optical parameters in the model.

		**n_{eff}**	
		**PC**	**PC-G**
METHOD 1 (Fitted experimental data)	$\lambda_B^2 = 4d_{hkl}^2\left[n_{eff}^2 - sin^2\left(asin\left(\frac{sin\theta_i}{n_{eff}}\right)\right)\right]$	1.26	1.34
METHOD 2 (Fitted experimental data)	The intercept, $I = (1.633\ D)^2 n_{eff}^2$ of the linear graph as in Figure S6	1.28	1.33
METHOD 3 (Calculated)	$n_{eff} = \left(n_p^2\phi_p + n_w^2\phi_w + n_G^2\phi_G\right)^{1/2}$	1.45	1.46
METHOD 4 (Ellipsometry)	$n_{eff}(\lambda) = \mathrm{Re}\left\{\tan\theta\sqrt{1-\frac{4\rho\sin^2\theta}{(\rho+1)^2}}\right\}$	-	1.33-1.41
